# Surgery

**Published:** 1895-09

**Authors:** 


### SURGERY.

EDITED BY FLOYD W. MCRAE. M.D.

### THE FINAL RESULTS IN RADICAL OPERATIONS FOR REDUCIBLE HERNIÆ.

Beresowsky (*Deut. Zeitschr. für Chir.*, 1895, Band xxxix. Hefte 3 und 4), after studying the various methods and describing the one he now favors, concludes his valuable study with the following summary:

1. The indications for operation, which during the past year (1892–93) have been held in the Berne Clinic, are confirmed by the last two hundred and twenty operations. The wish of the patient may be considered a sufficient indication, since the mortality is *nil* and the percentage of relapses is very slight.

2. The size and the age of the hernia have an influence on the period of recovery, and in most cases with regard also to recurrence.

3. The age of the patient—*i. e.*, the laxness of the abdominal walls—influences in no manner the result of the operation or the rapidity of the healing; it has a slightly greater influence upon the prognosis as regards recurrence. In case of operation for inguinal hernia under the above conditions a relapse of the original hernia is not so frequently observed as a return in some other locality which has no relation to the former area, and is apparently due entirely to the relaxation of the abdominal parietes.

4. Thanks to the good course of the operation and wounds, the operation for oblique inguinal hernia can be performed upon children at an early age.

5. The best method for performing the radical cure for oblique inguinal hernia, the author considers, is the last manifestation of Kocher's method, since, in the first place, it produces not less certain results, as far as recurrence is concerned, than the already known best methods (Macewen, Bassini); and, secondly, since this method, on accoont of the simplicity of its technique and the harmlessness to the patient, if there should be any accident in the process of healing, has decided advantages.

6. In order to prevent recurrences it is very necessary, during the operation, to observe the condition of the spermatic veins, and in case a varicocele is ever produced, it should be operated upon as speedily and thoroughly as possible.

7. The wearing of a truss after a properly performed operation, followed by primary union, is entirely unnecessary, since the majority of the patients—laborers—sent out from the clinic remain free from relapse without wearing any form of truss or protection over the wound.—*Am. Jour. Med. Sciences.*

---

### SERUM THERAPY FOR SYPHILIS.

The first trial of the serum therapy was made by P. Tommasoli. "Syphilis," says he, "is a disease exclusively human; all trials of inoculation upon the lower animals have failed. It is, therefore, allowable to suppose that in the blood of these animals there exists a chemical substance which renders them refractory, either preventing inoculation or rendering impossible the reproduction of the virus." By inoculating man with the serum drawn from the blood of these animals a special resistance is conferred.

At almost the same time Pellizari gave voice to his ideas upon the subject, and he took an altogether different view of the matter. Pellizari arrived at the conclusion that the products secreted by the syphilitic microbe have an antagonistic action upon the microbe itself. With this idea in view, he endeavored to introduce, into the organism of the syphilitic, serum taken from another syphilitic, and containing, therefore, the syphilitoxin.

In Tommasoli's experiments, serum was taken from the lamb, the dog, or the rabbit, and injected under the skin of the buttocks in doses varying from 2 to 8 c.c. at certain intervals. The results were satisfactory. In one case, a woman, twenty-six years old, who presented the lesions of syphilis in marked degree, a cure was apparently effected in twenty days. The reactions which occurred were varied in character. The most notable of these were rise in temperature, malaise, headache, gastric disturbances, or great weakness. At times an induration, redness, or urticarial eruption around the part injected occurred. In three cases shock followed, but all of the unpleasant symptoms were of short duration. Relapse had not occurred in a period of four to seven months. Other authors confirm Tommasoli's results, while some have had negative results from their experiments. Pellizari administered the serum in doses of 0.5 to 1 c.c. every third day at first, and later every day. He notes a rapid diminution of induration in the syphilitic lesions, and, perhaps, attenuation of the general symptoms. Mazza has modified Pellizari's method by taking serum from an animal which has been inoculated with the blood of a syphilitic. His reasons for so doing are not succinct, but, as he says, all experiments are justifiable. Conclusions cannot yet be made upon this subject.—*American Medico-Surgical Bulletin.*

---

### ANTIPYRIN IN SURGERY.

In the *Philadelphia Medical News* Dr. Roswell Park again calls attention to the value of antipyrin as a styptic. He has made frequent use of it since 1885, and does not hesitate to pronounce it the most valuable styptic we have, and an antiseptic as well, comparing "very favorably in this respect with most of the aniline or coal-tar derivatives that we use in medicine." He employs it most frequently in a five-per cent. solution, in sterilized water, as a spray, and does not hesitate to use it in the peritoneal cavity, or upon the surface of the brain. He says: "I have found that antipyrin has power, not sufficient to contract vessels of any size that spurt, but to almost instantly blanch and check oozing from any surface from which blood is escaping just fast enough to be an annoyance." . . . . "It may be injected into a cavity, as in bone; it may be applied on compresses to the oozing surface, or it may be sprayed as mentioned; but no matter how applied, under circumstances indicated within the limits given, it will seldom, if ever, be found to disappoint." He has found it, bacteriologically and in practice, antiseptic, and it is practically unirritating. It is especially valuable in operations within the nose, mouth, urethra, and bladder.

The remarkable analgesic properties of antipyrin, locally applied in inflammation of mucous membranes, have been pointed out by Hinkel (*New York Medical Journal* for October 28, 1888), Vigneron (*Concours Med.*, August 11, 1894), and others. We shall be glad to have reports from other surgeons who have used it, and especially pleased to know if it can be used without harm about the eyes. If so, it will prove a great boon to the ophthalmologist in some operations upon the eye and its appendages, which are often rendered very tedious and difficult by the presence of even a small quantity of blood.—*The Railway Surgeon.*

---

### INSOMNIA IN SURGERY AND ITS TREATMENT.

Van Schaick (*New York Medical Journal*, March 2, 1895) directs attention to the various forms of insomnia met with in surgical practice. The fear of an impending operation, though no longer so intense as in preanesthetic days, is yet an important factor in some cases, and hence requires treatment. The author knows of no better hypnotic for this purpose than trional given in a 15-grain dose, followed up if necessary by another one within an hour. This drug is preferred on account of its comparatively rapid action, of its lack of after-effects, and of the deep sleep it induces. It has also been found very useful in cases of depreciated nervous condition due to exhausting diseases and pain, in which chloral, though a more effective sleep-producing drug, is contraindicated from its action as a heart depressant. Restlessness and jactitation occurring after an operation without serious shock—a condition probably due to relaxation of a highly-strung nervous condition—may also be relieved by trional in moderate doses. Abdominal section, in which thirst is apt to complicate the other nervous disturbances, is also very frequently followed by insomnia of a more or less severe type. In two instances of this kind trional given by the rectum has, in the author's hands, proved quite successful. This drug is further recommended in cases of insomnia due to certain pathological nervous conditions which may complicate surgical disorders—such, for instance, as alcoholism, arterio-sclerosis causing intracranial headache, insanity, tabes, and other diseases of the cord, and the results of the abuse of narcotics.—*Brit. Med. Jour.*

---

### THE ASEPTIC TREATMENT OF SUPPURATION.

Zeidler (*Cent. für Chir.*, April 6, 1895) commends asepsis in the treatment of suppurating wounds. The method employed is the following: The field of operation is carefully prepared. After the ordinary operative technique the cavity is wiped out with sterilized gauze. As a rule, irrigation is not necessary; if it seems necessary, a sterile 0.6 per cent. salt-solution should be used. The wound is then packed fully with sterilized gauze, but not tightly. The further dressing is of sterilized absorbent material.

At the next dressing the skin about the wound is washed with ether or benzine, and the granulating wound dressed with dry sterilized gauze without irrigation. In most cases a dry dressing is sufficient; in some, however, a moist dressing does better; the moistening should be done with salt-solution. The author has not seen decomposition in the wound-secretion in a dressing that remained on for eight days. Free drainage with plenty of sterile absorbent dressing is all that is requisite. The course of the healing is very good. The amount of secretion is small. The dressing is renewed every eighth day.—*Am. Jour. Med. Science.*

